# Editorial: Health system response to the coincidence of the COVID-19 pandemic and disasters: a call for action

**DOI:** 10.3389/fpubh.2023.1281042

**Published:** 2023-09-15

**Authors:** Sanaz Sohrabizadeh, Luis Mockel, Mohammad H. Yarmohammadian, Mehdi Zare

**Affiliations:** ^1^Air Quality and Climate Change Research Center, Shahid Beheshti University of Medical Sciences, Tehran, Iran; ^2^Department of Health in Emergencies and Disasters, School of Public Health and Safety, Shahid Beheshti University of Medical Sciences, Tehran, Iran; ^3^IU Internationale Hochschule GmbH, University of Applied Sciences, Düsseldorf, Germany; ^4^Health in Disasters Department, Health Management and Economics Research Center, Isfahan University of Medical Sciences, Isfahan, Iran; ^5^World Business Institute at the US (WBIUS), Fairfax, VA, United States; ^6^Earthquake Prediction Center and Engineering Seismology Department, International Institute of Earthquake Engineering and Seismology (IIEES), Tehran, Iran

**Keywords:** disasters, COVID-19 pandemic, health system, lessons learned, epidemics

The COVID-19 epidemic started in the Chinese city of Wuhan in December 2019 (1). According to the last classification of the Center for Research on the Epidemiology of Disasters (CRED) published in EM-DAT, epidemics have been categorized as biological disasters (2) and need urgent preparedness and response actions (3). In the case of COVID-19, healthcare systems across the world tried to manage this pandemic in an effective way. However, new problems and challenges appeared and worsened the situation of the affected communities at the time of the COVID-19 pandemic response. Of these emergent challenges, natural disasters (e.g., earthquakes, floods, and tornadoes) co-occurring with the pandemic were the most important and seriously affected the capacities and capabilities of health systems in several countries (4). Natural disasters have considerable health, social, and economic effects by themselves and their co-incidence with the COVID-19 epidemic imposed multiple pressures and complicated conditions on healthcare systems. Furthermore, the co-incidence of natural disasters and epidemics can significantly influence the vulnerabilities and capacities of health systems and put people's health at high risk. Therefore, facing the dual risk of disasters and epidemics (e.g., COVID-19) can be a vital issue for health systems and needs to be studied and focused on by researchers, scholars, and health system administrators. Thus, the Research Topic “*Health system response to the co-incidence of epidemics and disasters: the importance of lessons learned during the COVID-19 pandemic*” was selected and notified by the journal “*Frontiers in Public Health*” to publish research papers that investigated and reported the different aspects of the health system response to the co-incidence of the COVID-19 pandemic and disasters across the world.

A total of 11 papers related to this Research Topic were accepted and published in the journal. None of the submitted papers addressed the co-incidence of the COVID-19 epidemic and natural disasters but considered the COVID-19 epidemic as a disaster and reported its various health aspects. For instance, regarding the social health aspect of COVID-19, social vulnerability indicators were developed to highlight the incidence of the COVID-19 epidemic in socially vulnerable regions in Iran (Moslehi et al.). People's risk perception regarding the COVID-19 pandemic was investigated by Chinese researchers. They indicated that the older population, as a vulnerable group, had the highest risk perception score regarding the COVID-19 epidemic in China (Chen C. et al.). Several authors investigated and analyzed the status of healthcare providers at the time of COVID-19. For example, the insufficient preparedness of nurses to respond to the COVID-19 crisis in Iran was extracted and reported (Moradian and Mahmoudi). In Germany, the self-images of ICU clinicians for crisis management during the COVID-19 epidemic were explored and reported as important perceptions that should be notified by policymakers (Piel et al.). NGOs, as one of the important relief providers, can supply emergency relief resources with the support of the government through media publicity and policymaking encouragement in China (Lu and Wang). Regarding post-disaster recovery, resilient communities could cope with the effects of the COVID-19 epidemic better than vulnerable communities, which have insufficient communications and information (Irasanti et al.). The surge capacity of healthcare facilities was an important issue at the time of the COVID-19 epidemic. Accordingly, the Arizona Department of Health Services has designed and implanted a surge line since the COVID-19 pandemic. This line was successful in facilitating patient transfer and benefiting marginalized populations (Villarroel et al.). Medical performance evaluation and COVID-19 vaccination optimization were also investigated by Chinese authors, who published their research in this Research Topic (Chen M. et al.; Wen et al.).

Although the impacts of disasters and epidemics on health systems have been reported by several studies, little is known about how to reduce the dual risk of disasters and epidemics. The COVID-19 epidemic provided a unique opportunity for identifying the emergent challenges and establishing innovative risk reduction and preparedness strategies to respond to similar crises in the future. The health sector has the main responsibility for people's health, and thus, plays special roles during the co-incidence of epidemics and disasters. Applying the lessons learned during the COVID-19 epidemic and establishing resilient health facilities and prepared healthcare staff can reduce deaths, injuries, and health system destruction after epidemics and disasters. Otherwise, all the adverse challenges and issues that the world experienced during the COVID-19 pandemic will probably repeat in any similar pandemic in the future. Thus, health systems need scientific collaborations and policymaking efforts at regional and international levels. The documentation and publication of effective lessons learned by health systems in the areas of prevention and treatment and disaster and emergency management should be considered as important measures and the first steps in future planning and policymaking.

## Author contributions

SS: Writing—original draft, Writing—review and editing. LM: Writing—review and editing. MY: Writing—review and editing. MZ: Writing—review and editing.
